# Echinocandins and Their Activity against Aspergillus terreus Species Complex: a Novel Agar Screening Method

**DOI:** 10.1128/aac.01909-21

**Published:** 2022-02-15

**Authors:** Nina Lackner, Roya Vahedi-Shahandashti, Sonja Jähnig, Lea Schönherr, Cornelia Lass-Flörl

**Affiliations:** a Institute of Hygiene and Medical Microbiology, Medical University Innsbruck, Innsbruck, Austria

**Keywords:** *Aspergillus terreus*, agar screening method, antifungal susceptibility testing, echinocandins

## Abstract

We evaluated the newly proposed agar screening method for echinocandin susceptibility testing of 144 *Aspergillus* section *Terrei* isolates compared with the Etest method. Both methods defined the isolates to be wild-type strains for anidulafungin and micafungin, with Etest minimal effective concentrations (MECs) of ≤0.004 mg/L. For caspofungin, the novel agar screening method identified 37 isolates to be caspofungin non-wild type based on their fluffy colony appearance on caspofungin agar. Etest MECs for caspofungin for these isolates were scattered widely from 0.002 to 0.750 mg/L, showing only partial accordance between the two methods.

## INTRODUCTION

The frequency of life-threatening *Aspergillus* infections has increased over the past few years, contributing to high morbidity and mortality (1, 2). The emerging threat posed by Aspergillus terreus species complex is a matter of significant concern, as it shows a high tendency to disseminated infections and reduced susceptibility to amphotericin B and occasionally to azoles (3–6). In refractory cases, echinocandins are recommended as an alternative therapy for invasive aspergillosis (IA) (7). Echinocandins inhibit the synthesis of β-(1,3)-glucan, leading to a lack of cell wall integrity (8). For echinocandin antifungal susceptibility testing, broth microdilution (BMD) techniques have been standardized by the European Committee on Antimicrobial Susceptibility Testing (EUCAST) and the Clinical and Laboratory Standards Institute (CLSI); however, breakpoints are still missing (9, 10). Further, BMD methods are labor-intensive and sometimes difficult to interpret for filamentous fungi, which might lead to an underestimation of echinocandin resistance in *Aspergillus* spp. (11). Attempts to improve BMD techniques are being made, such as using the tetrazolium salt XTT (2,3-bis-[2-methoxy-4-nitro-5-sulfophenyl]-2H-tetrazolium-5-carboxanilide salt) for a colorimetric and therefore simpler readout (11). However, these methods are not yet included in the current EUCAST guidelines. For this reason, many laboratories rely on the application of agar-diffusion tests, which are commercially available as Etest and are easy to apply.

In the present study, we aimed to assess the susceptibility profile of anidulafungin, caspofungin, and micafungin against A. terreus species complex (*n* = 144) with the new agar-based method developed by Meletiadis et al. (12) and compare the data with that of the Etest method. This novel agar screening method (NASM) has been proposed but not yet published as EUCAST guideline 10.2 (personal communication). For control, susceptibility of selected strains was further tested according to the current EUCAST 9.3.2 protocol (9).

## RESULTS AND DISCUSSION

Etest of the control strain SSI-1794 resulted in MECs of 0.5 to 1, >32, and 0.25 to 0.75 mg/L for anidulafungin, caspofungin, and micafungin, respectively. Further, NASM showed equal fluffy growth in the antifungal containing and control wells after 24 h as well as 48 h (12). BMD according to EUCAST 9.3.2 for SSI-1794 resulted in an MEC of 16 mg/L for caspofungin.

**FIG 1:**
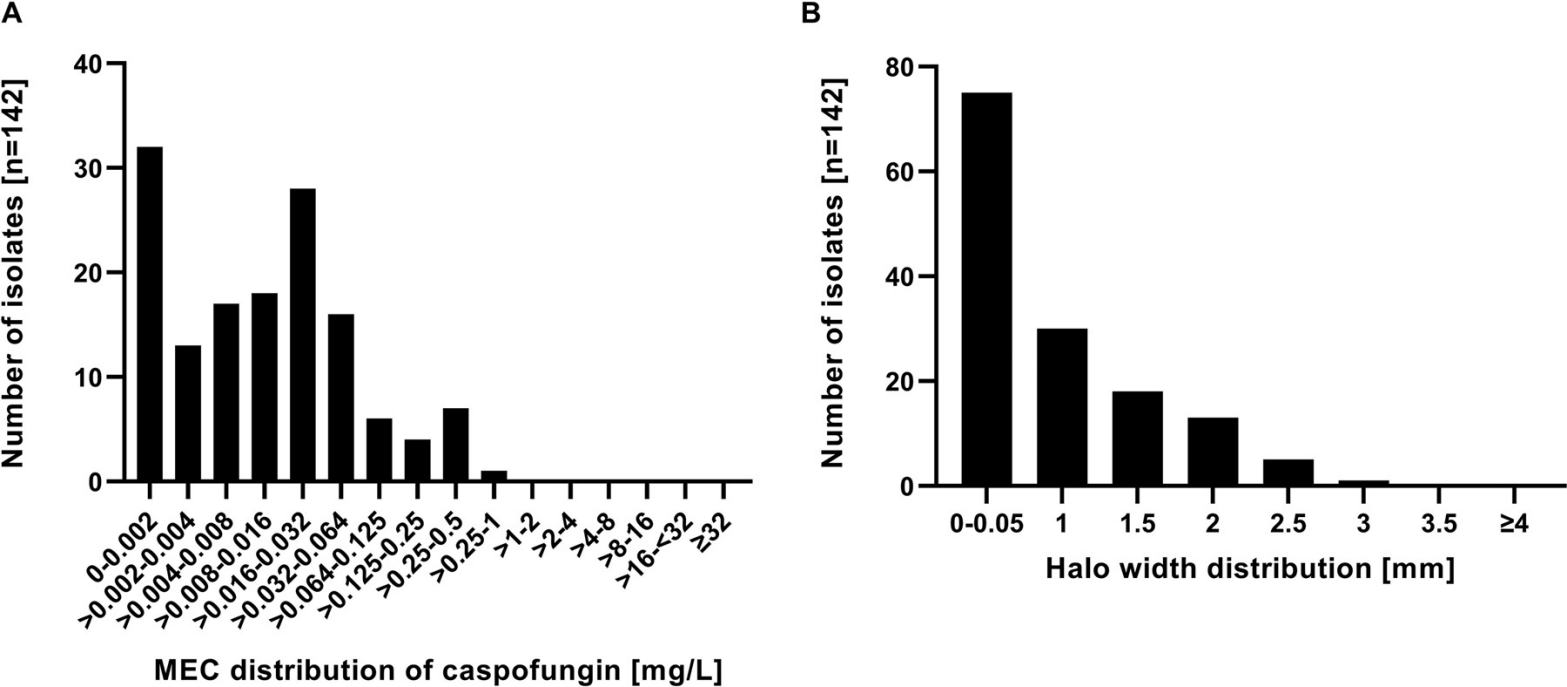
Susceptibility testing of clinical *Aspergillus* section *Terrei* isolates against caspofungin (means of technical duplicates). (A) Minimal effective concentration (MEC) distributions according to the Etest method at 37°C. (B) Width of halo on agar containing 1 mg/L caspofungin.

Two of the 144 *Aspergillus* section *Terrei* isolates showed insufficient growth in the control wells of the screening plates and were therefore excluded from the analyses. MECs according to Etest for anidulafungin and micafungin were ≤0.004 mg/L for all isolates. The MECs for caspofungin ranged from 0.002 to 0.750 mg/L (Fig. 1A). Higher MECs for caspofungin than for the two other echinocandins were also observed by Imbert et al., who found an MEC range of 0.016 to 0.25 mg/L in 79 clinical *Aspergillus* section *Terrei* isolates (13).

Agar plate assays were evaluated at two time points. After 24 h, at least weak fungal growth could be observed in all samples; however, it was not suitable for reading susceptibility results in all of the isolates. Therefore, we concentrated our analysis on the 48-h results, although Meletiadis et al. (12) recommend 24 h, if growth was sufficient. It should be mentioned that A. terreus strains grow generally slower at 37°C than Aspergillus fumigatus strains, explaining this discrepancy in optimal incubation time (5). According to the NASM (12), plates should be read by distinguishing between fluffy (morphology similar to the control) and nonfluffy growth, while ignoring small or irregular halos. While we found no isolates that met these criteria for anidulafungin and micafungin, 37 corresponding isolates for caspofungin were detected (Table 1). The median values of the MECs from the Etest were higher in these 37 isolates. However, single data scattered widely (Table 1). This means that an accordance between the NASM and Etest for single isolates cannot be presumed using these distinction criteria.

**TABLE 1:** Caspofungin susceptibility according to a novel agar screening method of *Aspergillus* section *Terrei* isolates compared with Etest minimal effective concentration results

Parameter	Nonfluffy colonies	Fluffy colonies, no small^*a*^ colonies or irregular halos
No. isolates meeting the criteria/no. total isolates (%)	105/142 (74)	37/142 (26)
Classification	WT	Non-WT
Species^*b*^		
Aspergillus citrinoterreus	28/36 (78)	8/36 (22)
Aspergillus terreus *sensu stricto*	61/88 (69)	27/88 (31)
Aspergillus alabamensis	7/8 (88)	1/8 (12)
Aspergillus hortai	11/12 (92)	1/12 (8)
MEC Etest (mg/L), median (min–max)	0.012 (0.002–0.250)	0.032 (0.002–0.750)

a ≤8 mm after 48 h.

b Values indicate no. isolates meeting the criteria/no. total isolates (%).

These results would indicate a surprisingly high rate of caspofungin non-wild-type (non-WT) *Aspergillus* section *Terrei* isolates. However, Meletiadis et al. also found WT A. terreus strains, which were unable to produce strictly nonfluffy colonies in caspofungin wells during their multicenter evaluation (12). They further stated that this irregular growth could still be morphologically distinguished from real fluffy growth. Unfortunately, we were not able to confirm this phenotype. The widths of the halos in the caspofungin wells showed a continuous right-skewed distribution (Fig. 1B), for which a threshold would have to be defined to ensure an unbiased readout. As we observed different growth rates for different isolates, this threshold should include the width of the halo of the control. Therefore, we decided to assess our data additionally using the widths of the halos in the echinocandin wells relative to the control. Only two of the 37 isolates, which were classified as caspofungin non-WT, showed halo widths similar (≥75%) to the control after 48 h, exhibiting mean MECs in Etest of 0.315 and 0.440 mg/L.

**FIG 2:**
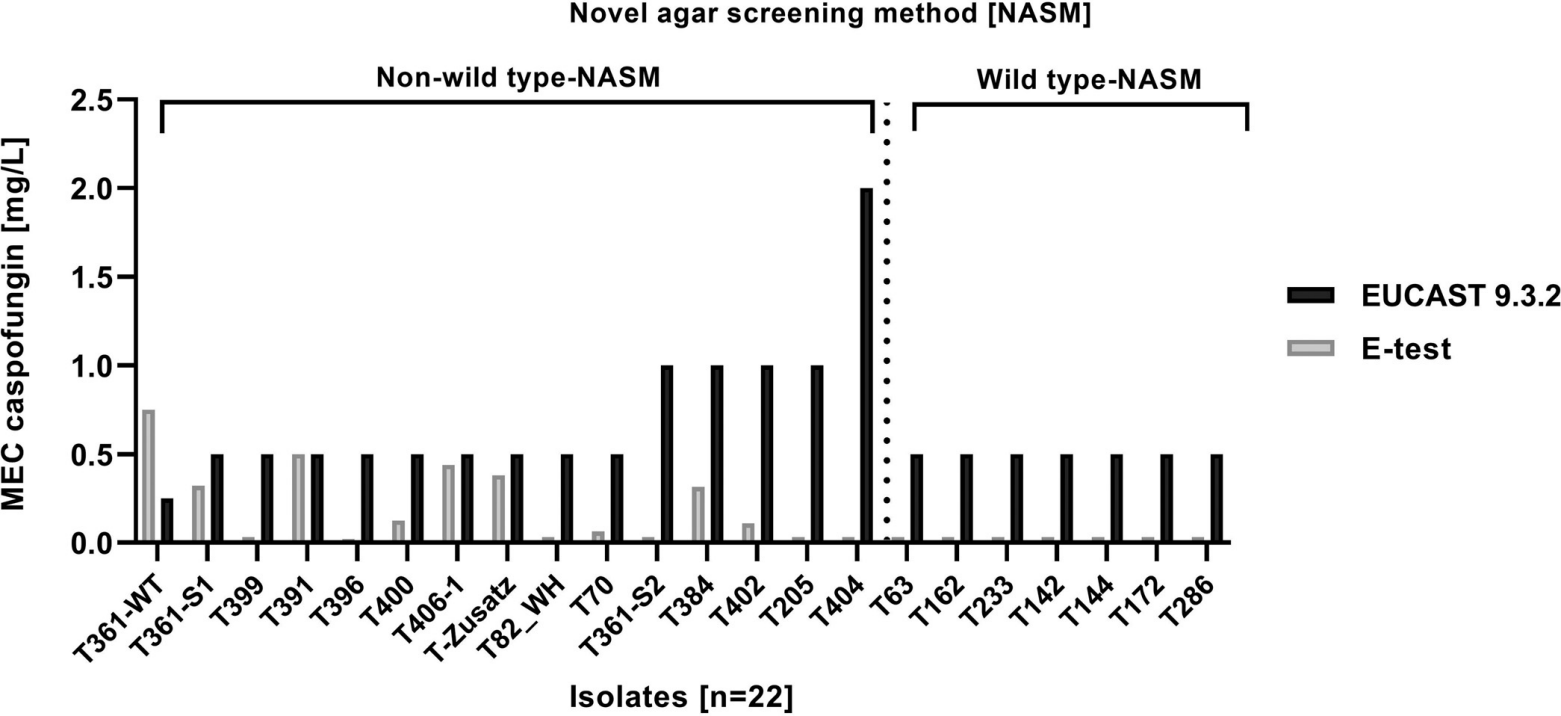
Minimal effective concentration (MEC) of caspofungin determined with Etest (values lower than the lowest concentration in BMD of 0.03 were rounded to 0.03) and broth microdilution according to EUCAST 9.3.2 of *Aspergillus* section *Terrei* isolates categorized as non-wild type or wild type by the agar screening method.

The comparison of susceptibility data from all three methods, NASM, Etest, and EUCAST 9.3.2, for a selection of isolates showed no agreement between EUCAST 9.3.2 and the other two methods (Fig. 2). In general, EUCAST 9.3.2 MECs were high with a median of 0.5 mg/L even for the NASM WT isolates. In this context, Imbert et al. (13) also found higher MEC values for EUCAST BMD compared with Etest when testing 79 clinical *Aspergillus* section *Terrei* isolates. Further, results for EUCAST BMD results for caspofungin and A. terreus varied up to two 2-fold steps, between 0.125 and 0.5 mg/L, in the multicenter comparison of Meletiadis et al. (12). This means that the reproducibility of BMD tests is too low to detect small differences in susceptibility between different *Aspergillus* isolates, highlighting once more the difficulties of echinocandin susceptibility testing for aspergilli.

Additionally, we assessed the formation of conidiophores in the screening wells. Although we could generally observe a delayed production of conidiophores in the echinocandin wells compared with the control, there were no differences between the isolates classified as caspofungin WT and non-WT; hence, this observation does not result in an additional value.

Compared with Etest, the consumable costs are much lower for the NASM, but it is more time-consuming. Further, the interpretation of the results is more subjective than for Etest because there can be a smooth transition from nonfluffy to fluffy colonies. To overcome this problem, the size of the halo could be taken into account, introducing thresholds for the ratio of halo width between echinocandin and control wells. Further studies are needed to evaluate this approach. Another drawback of the method is the use of only one concentration for each echinocandin. This can cause problems, especially for isolates that show MIC phenomena. Our conclusion is that it should be evaluated if the test concentrations of the antifungals chosen for A. fumigatus non-WT isolates are also appropriate for non-WT isolates of other *Aspergillus* species.

## MATERIALS AND METHODS

**FIG 3:**
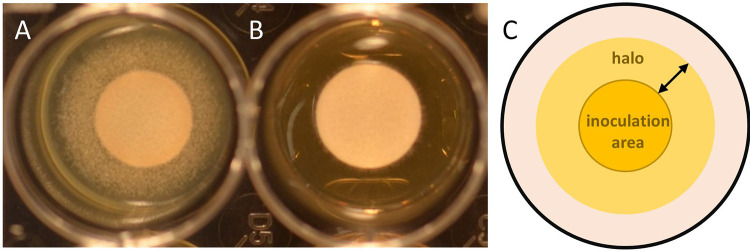
Fluffy (A) versus compact (B) growth of *Aspergillus* section *Terrei* and scheme defining the halo width (C) in an agar-containing well of the novel agar screening method (12).

We examined 144 clinical isolates of *Aspergillus* section *Terrei* from our international strain collection, including 36 Aspergillus citrinoterreus, 12 Aspergillus hortai, 8 Aspergillus alabamensis, and 88 A. terreus *sensu stricto*. The A. fumigatus mutant SSI-1794 served as control and appeared to be a non-wild-type (non-WT) strain for echinocandins (14). For NASM, echinocandin-containing as well as echinocandin-free RPMI 1640 (Sigma-Aldrich, USA) agars were prepared and aliquoted (0.5 mL/well) row-wise in 24-well plates according to Meletiadis et al. (12). The concentrations tested were 0.25, 1, and 0.125 mg/L for anidulafungin, caspofungin, and micafungin, respectively. Each well was inoculated with 20 μL spore suspension (McFarland standard of 1 to 2) and incubated at 37°C for 48 h. Tests were performed in technical duplicates and read after 24 and 48 h. Well plates were analyzed assessing the morphology (fluffy versus nonfluffy growth) (Fig. 3a and b), and categorized as wild type (WT) and non-WT according to the NASM (12). Additionally, we determined the width of the halos (Fig. 3c) as well as the abundance of conidiophores using a reflected light microscope (categories included many, some, or no conidiophores).

Etest was chosen as the main control method because it is widely used in routine laboratories, easy, and timesaving. Further, Etest and NASM are both agar based, which enables similar growth of the fungi and may lead to a higher comparability between the methods. For Etest (bioMérieux, Marcy-l’Étoile, France), the spore suspensions were diluted (McFarland standard of 0.5) and inoculated on RPMI 1640 (Sigma-Aldrich, USA) agar plates with 2% glucose in technical duplicates. Subsequently, the assays were incubated at 37°C for 24 h until analysis according to the manufacturer’s instruction. In case of poor growth, plates were incubated for an additional 24 h at room temperature. Minimal effective concentrations (MECs) for Etest were defined as the transition line between normal and scarce growth.

Finally, we performed BMD for caspofungin susceptibility testing according to EUCAST 9.3.2 guidelines (9) for a selection of isolates, comprising the A. fumigatus mutant SSI-1794 and 15 non-WT and 7 WT *Aspergillus* section *Terrei* isolates defined by NASM. Susceptibility testing against anidulafungin and micafungin was not performed using BMD due to the lack of non-WT strains for these antifungals.
